# Immersion Therapy with Head-Mounted Display for Rehabilitation of the Upper Limb after Stroke—Review

**DOI:** 10.3390/s22249962

**Published:** 2022-12-17

**Authors:** Klaudia Marek, Igor Zubrycki, Elżbieta Miller

**Affiliations:** 1 Department of Neurological Rehabilitation, Medical University of Lodz, Milionowa 14, 93-113 Lodz, Poland; 2 Institute of Automatic Control, Lodz University of Technology, Stefanowskiego 18, 90-537 Lodz, Poland

**Keywords:** head-mounted display, virtual reality, rehabilitation, stroke, upper limb

## Abstract

Immersive virtual therapy technology is a new method that uses head-mounted displays for rehabilitation purposes. It offers a realistic experience that puts the user in a virtual reality. This new type of therapy is used in the rehabilitation of stroke patients. Many patients after this disease have complications related to the upper extremities that limit independence in their everyday life, which affects the functioning of society. Conventional neurological rehabilitation can be supplemented by the use of immersive virtual therapy. The system allows patients with upper limb dysfunction to perform a motor and task-oriented training in virtual reality that is individually tailored to their performance. The complete immersion therapy itself is researched and evaluated by medical teams to determine the suitability for rehabilitation of the upper limb after a stroke. The purpose of this article is to provide an overview of the latest research (2019–2022) on immersive virtual reality with head-mounted displays using in rehabilitation of the upper extremities of stroke patients.

## 1. Introduction

In recent years, rehabilitation using virtual reality has become increasingly popular. VR technology offers capabilities based on human interaction in a simulated environment that receives continuous feedback on performance and efficiency [1]. For effective neurological rehabilitation, training should be challenging, repetitive, task-specific, motivating, relevant, and intense to activate neuroplastic mechanisms in the brain [2]. Virtual reality technology seems to meet all of the above requirements. VR immersive therapy, in which patients receive a wireless HMD per head, enables attendance in a fully simulated environment. This allows the user to experience the illusion of being in a computer-generated environment instead of in a real-world environment in real time [3]. The system allows patients to perform actions while being immersed in virtual games [4]. The level of immersion is difficult to estimate by users. It indicates the degree to which a person exists in a reality made by technology [5,6,7].

Stroke is the second most common cause of death and the third cause of disability in the world. The recorded increase in the incidence of stroke is a serious public health problem, but also a problem of social and economic development of society [8]. Stroke is the second leading cause of death and the leading cause of disability in adults in the European Union [9]. This disease affects up to 1.1 million people [10] and causes about 440,000 deaths [11]. About 60–80% of stroke survivors have complications associated with the upper limbs [12]. Only 20% of people who have experienced severe hemiparesis return to full upper limb function, compared with 80% who have experienced mild hemiparesis [13]. As a matter of fact, rehabilitation after stroke is a crucial element of recovery that gives patients the possibility to regain their loss in abilities and independence [14].

In the review, Guzsvincz, Szucs, and Sik-Lanyi divided reality into virtual, augmented, and mixed reality. Virtual reality is a synthetic environment in which interactions can take place. Augmented reality expands into our reality, as the name suggests, while mixed reality allows interaction with extended objects [15]. The term “immersion” is often used in the context of virtual reality. Immersion is the degree to which the user feels present in a virtual reality rather than in the real environment [16]. Non-immersive virtual reality allows users to interact and observe using devices that are not able to fully reproduce sensory perception [17]. The VR user can control the environment and receive the generated stimuli (images, sound, touch). A person using this form of virtual reality may feel less in the virtual environment as they interact with the content of the generated reality on a screen. Interaction with objects in a virtual environment is possible in 2D or 3D modes. Non-immersive VR systems are usually equipped with a computer, a monitor, a mouse, and a controller [18,19].

**Figure 1:**
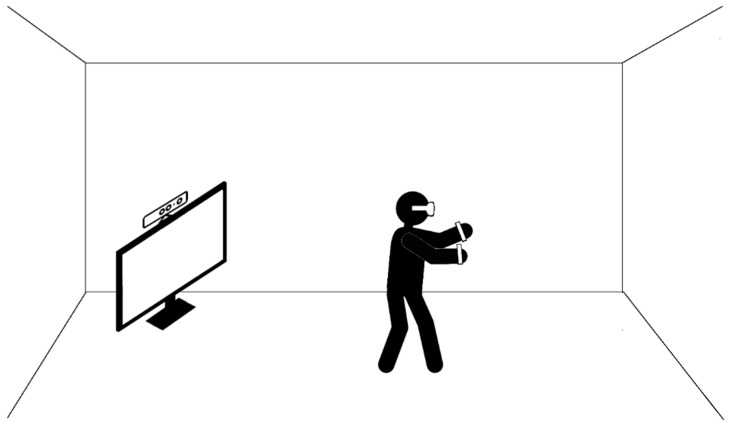
Simplified illustration of patient performing immersive virtual reality therapy.

**Figure 2:**
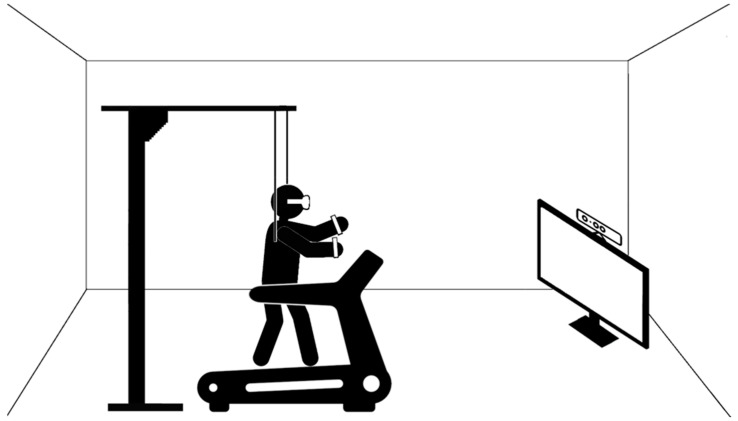
Simplified illustration of a patient performing immersive virtual reality therapy with a treadmill.

Immersive VR enables a more comprehensive virtual reality experience in a three-dimensional environment. Computer equipment provides visual and acoustic visualization, which can also be reflected in the tactile interactions of the user. This makes it possible to experience the illusion of being in an imaginary environment that is not visible on the screen. Users who use this form of VR may need a special headband (HMD), but also can use haptic devices, motion trackers, data gloves, and wireless controllers (Figure 1). Visual-based tracking and imaging systems like IREX can also be used. They allow participants to participate in different activities in a simulated environment. Other necessary equipment are inertial sensors: gyroscopes and accelerometers, which are responsible mainly for tracking the movement of the head [20]. The cost of the hardware that enables interaction with the virtual environment varies greatly. Total immersion VR requires an artificial simulator that offers several types of experiences. Non-experimental VR therapy is cheaper, considering that immersion VR therapy can also extend the therapy with physical objects placed in space. The user can perceive their texture and even their temperature. In addition, it is possible to purchase VR treadmills for immersion therapy, allowing users to run or move in place (Figure 2). This tool solves the frequent problem of lack of space for this type of therapy [3,21,22,23]. 

Neurological rehabilitation, consisting of platforms, cameras, and sensors used for virtual reality games, is well-tolerated by patients and arouses curiosity. Some studies show that conventional therapies appear monotonous and boring to patients [24,25]. This shows that neurological rehabilitation needs to be varied. Therapy with modern technologies increases the motivation of patients [26,27,28,29]. Several elements can help to increase motivation: game mechanics (including scoring), results feedback (including leveling up), and game fun [18]. Previous virtual reality interventions have brought benefits to patients with movement disorders due to neurological diseases. The behavioral and neurophysiological benefits of motion observation [30], imaging [31], and repetitive exercises [32] are well-known. Virtual reality therapy has an enormous potential for the functional regeneration and plasticity of the brain. An increased potential occurs at the beginning of the recovery phase, and neuroplastic changes in the brain are made possible by the simulation of sensorimotor areas. This enables the compensation process in the body [33]. Sensorimotor cortex reorganizations are confirmed by magnetic resonance imaging [34].

The load on the neck and head when using the head-mounted display is an important criterion when choosing therapy for neurological patients having complications in the upper limbs and balance disorders. The use of an HMD causes changes in locomotion and postural control by manipulating optical flow [35]. Neck strain is an important indicator for HMD application [36]. When using an HMD, patients are forced to constantly change their body position in the virtual environment. This strains the musculoskeletal system, especially the head and neck area [37]. Around the neck, torque is formed. Greater fatigue and discomfort may occur in people using heavier HMDs, exerting more pressure on the head. This may result in worsening rehabilitation outcomes, as the therapy may prove to be severe and physically burdensome [38]. Weight has a direct impact on the patient’s comfort when using an HMD, which is why Mehrfard el al. made a special set of metrics that should be taken into account when choosing a head-mounted display. These include weight, heat development, image quality, compatibility with glasses, and tracking stability [36].

In this review, we discussed rehabilitation using immersion therapy in post-stroke patients with upper limb complications, including: immersion therapy technology, clinical application and evidence of effective use in neurological rehabilitation, barriers that arise in the use of immersion therapy, and considerations for limitations and future use of immersion therapy among post-stroke patients with upper limb complications. This review provides an important contribution to insight into the effectiveness of immersive therapy rehabilitation in stroke patients. Immersion virtual reality therapy with the use of head-mounted displays gives the impression of the possibility of making rehabilitation more effective and engaging, due to the resulting motivation for a virtual reality with which patients interact [39]. Many previous published reviews of VR rehabilitation omit immersion therapy using head-mounted monitors, failing to show the potential of this type of therapy [40,41,42,43]. 

## 2. Materials and Methods

The material gathered for this review was sourced from the following databases: PubMed, PubMed Central, Cochrane Library, Embase, Medline, Web of Science, and SCOPUS. The topics searched for included:

- Immersive therapy upper limb stroke;
- Rehabilitation after a VR stroke;
- HMD stroke rehabilitation;
- HMD rehabilitation of the upper limb;
- HMD stroke.

**Figure 3:**
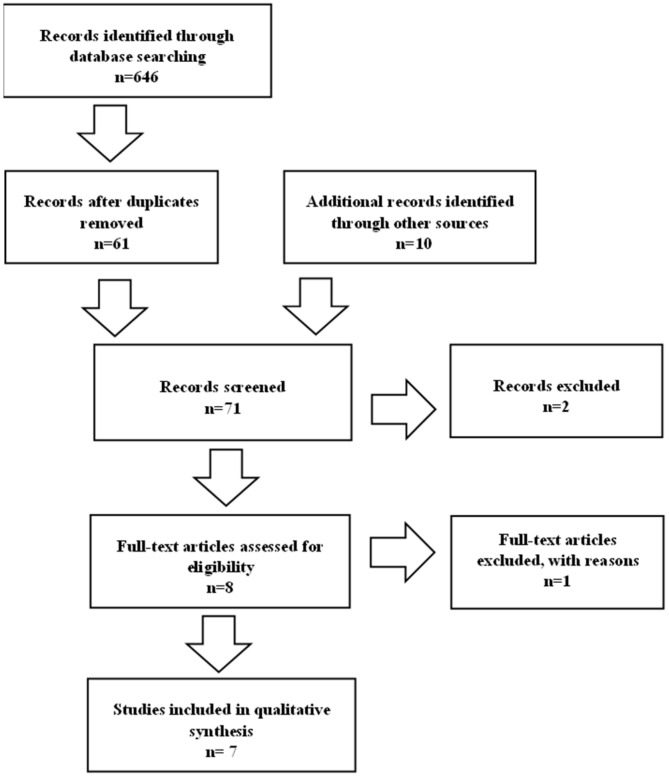
Flowchart of study selection.

In addition to the main issues, the abbreviations HMD and VR HMD were also searched. A total of 646 articles were analyzed. After removing the duplicates, 61 articles qualified as well as an additional 8 articles found by manually searching the reference lists. We excluded articles when: Head-mounted displays were not used for their rehabilitation; Rehabilitation was not used for the upper extremities; Rehabilitation was not for stroke patients; Rehabilitation with virtual reality was non-immersive or semi-immersive; Head-mounted displays were used for purposes other than rehabilitation of the upper extremities; Studies were published in a language other than English. Therefore, only 7 studies were approved to be studies included in our qualitative synthesis that met all requirements (Figure 3).

## 3. Results

Immerse VR is a relatively new form of neurological rehabilitation in patients with disorders in the upper limb after a stroke, which is why the number of studies in this area is limited. The research found from the last 5 years is presented in the tables below (Table 1 and Table 2).

The longest study involving patients lasted 12 weeks [50], the shortest 2 weeks [48,49]. Only two studies included more than 50 patients [44,50]. Four studies involved less than 20 patients in a research project [45,46,47,48]. One of them was a feasibility study [46].

All of the studies collected in the review used their own type of virtual games performed by patients to improve the indicators of the upper limb. Games that were used consisting of cooking, decorating trees with leaves and fruits, collecting vegetables, playing drums [44], climbing, shooting balloons [47], grabbing and transporting balls [48,49], collecting and arranging stones of various shapes, arranging plates, moving fruit from plates, or even setting the table [45]. 

Two studies conducted by Mekbib in 2020 [48] and 2021 [49] equipped two HTC Vive tracking base stations (lighthouses), which were responsible for the accurate tracking of patients’ locations in 3D.

The conducted studies presented their results as: an improvement in the function of the upper limbs [44,46,47,48,49,50], an improvement in functional independence derived from the improvement of the quality of everyday life [46,49], and satisfying assimilation of the system by patients [45]. Not all the factors studied were improved, for example, functional independence [44]. The authors mostly had a similar study design, using the internationally recognized scales or their modifications: Fugl-Meyer Upper Extremities, Box and Block Test, Action Research Arm Test, and Barthel Index. Four of the studies involved patients after two types of strokes (ischemic and hemorrhagic), two studies involving patients after ischemic stroke, and in one of them, no detailed classification [47] was given. Rehabilitation tasks using the head-mounted display lasted no longer than one hour in any case. 

Of the seven studies, only one study conducted by Hsu et al. investigated the occurrence of adverse reactions to HMD. The authors did not record any adverse events such as dizziness, visual disturbances, or injuries to the upper extremity [50]. This shows the high safety of the study.

None of the seven articles addressed the head-mounted display, which could affect posture control and strain on the head and neck. These limitations may have been relevant to the results obtained by patients during rehabilitation.

## 4. Discussion

The purpose of this review was to look for studies with neurological rehabilitation of the upper limb for people after a stroke, in which immersion therapy with a head-mounted display (HMD) is used. This offers a broader view of a new and still rarely used technology that could have potential benefits in future rehabilitation. Virtual reality provides a rich and customized motor training, thanks to intensity modulation and sensory feedback [51,52]. 

The studies considered in this review had small samples of subjects; some of them were feasibility and pilot studies [45,46]. This means that the therapy is still in the early stages of development and application. In the future, we can expect more published research using head-mounted displays (HMD). There are still no studies involving more than 100 stroke patients with upper limb dysfunction. A larger study would show the variety of results from the use of head-mounted displays in rehabilitation. VR systems engage the participant in tasks resembling everyday activities and using a lot of important movements for which the upper limbs are responsible, such as grasping, catching, and reaching [47,48]. They carry out unilateral and bilateral training of the upper limbs, affecting the regeneration and reproduction of impaired limb functions after a stroke [49]. It turns out that immersive therapy with the use of a head-mounted display (HMD) as a VR system is a suitable therapeutic tool not only from the point of view of rehabilitation, but also for better assimilation of skills and correct movement patterns and the ability to transfer skills acquired during VR tasks to everyday life. In the future, this will help to increase the self-reliance and functional independence of neurological stroke patients [44,49]. 

The use of immersive VR in neurological rehabilitation in patients with upper limb dysfunction after a stroke is still being studied. This is a relatively new advance in technology that is not as extensively researched as non-immersive VR [22]. This review included studies using HMDs (head-mounted displays) to immerse patients with upper limb dysfunction after a stroke in fully immersive virtual reality environments [44,45,46,47,48,49,50]. Some studies, in addition to studying the effectiveness of VR therapy, also focused on neuronal activation, performed MRI tests confirming changes in the cerebral cortex [48,49], performed mirror therapy [45,48,49,50], tested the usefulness and acceptance of [46] therapy, and simultaneously checked three different groups: conventional therapy, mirror therapy, and mirror therapy with an HMD as immersive VR therapy [50]. 

In recent years, many studies have been conducted using fully immersive environments for imbalances [53,54,55], gait teaching and postural control [54,55,56], or safety and fall risk [57]. These concerned post-stroke complications associated with the lower limbs. More and more tests are being performed aimed only at comparing conventional rehabilitation with rehabilitation using fully immersive VR therapy, while their number is still small [44,46,47]. The use of modern technologies are allowing virtual reality to be used more often in healthcare [58]. Patients using VR therapy show greater motivation to exercise [59,60]. In addition, virtual reality technology shows a positive attitude towards the strategy of coping with the disease, which only shows another benefit of modern technologies [61].

A major barrier of virtual reality rehabilitation for the elderly can be modern technologies with touch equipment, large screens, goggles covering the entire eyes, or even motion controllers. Such equipment, if patients have not previously shown interest in virtual technology, can be a novelty or even a misunderstanding, which affects the emergence of digital exclusion. In the study by Kamińska, Miller, Rotter et al., patients of mature age found that training in virtual reality technology was a way to stop the process of digital exclusion. The benefit of getting used to the virtual world allowed them to adapt to modern technologies [62]. 

The deployment of new digital services and platforms has been caused by the COVID-19 pandemic. Accelerated change can be a problem and a challenge for older and less prosperous people. The phenomenon of the digital divide is becoming increasingly noticeable. Divide is described as an incongruity between groups in which one group has the resources, the will, and the ability to use digital technologies, and in the other group, the capacity and resources for an advanced approach are lacking. In this group, lack of measures can lead to demographic and socioeconomic inequalities around the world [63,64,65]. There are also situations when older people do not have access to wireless internet at home, which is a barrier to expanding knowledge in the field of digital technology [66]. In such circumstances, it is conceivable that older people may be reluctant to use new technological developments, such as virtual reality technology designed to rehabilitate patients. More and more households can afford to purchase virtual reality systems and head-mounted displays [67]. Previously unknown, the new technological progress that patients may encounter in health facilities may contribute to reducing the digital divide, but not to its complete exclusion; however, the problem is still based on economic and social considerations.

Technology is constantly changing, which can pose a threat to the profession of physiotherapists, who indicate that they feel unprepared. There is a concern about whether all physiotherapists will cope with the progress of the digital and technological age [68]. Employees’ training should be the main rule when buying equipment for a health center. The new therapies using modern technologies, including VR, may be hindered by regulations and legislation determined by national regulatory authorities. Regulations of new procedures for introducing new technologies into clinical practice are held by the country. Telerehabilitation can be the example [68].

Like any other therapy, rehabilitation with the use of virtual reality also has its drawbacks, in particular, fully immersive therapy. Studies relating to the side effects of immersive VR therapy are mixed. Some of them have shown that using full immersion had a negative effect on static balance and was associated with side effects: headaches, dizziness, eye strain, and motion sickness [51,69]. In a study conducted in 2021 in which the occurrence of side effects was checked, there were no adverse events, nor any cases of motion sickness and dizziness. The therapy is positively received by patients and is safe [70]. The latest head-mounted displays are next-generation devices and symptoms of motion sickness are much less common [71]. Butt et al. decided to test HMDs on healthy students. The results were satisfactory, but there were side effects in the form of dizziness, nausea, and eye discomfort [72]. However, the possibility of side effects should be taken into account when choosing the type of rehabilitation for a neurological patient, which should be approached individually. 

Interest in virtual technology in rehabilitation is constantly growing. The first scientific articles on rehabilitation and virtual reality date back to 1962, according to the PUBMED research viewer. In 2022, 2040 works had been published under the keyword “VR rehabilitation”. The keyword “Immersion Head Mounted Display rehabilitation” has been used in 65 articles since 1998, which shows that more and more people from the medical world are no longer interested only in using non-immersive reality for the rehabilitation of patients. 

Rehabilitation with the use of HMDs in virtual reality does not seem to have the danger of losing interest in therapy among patients. In order to preserve the progress of technology and motivation among users, the level and variety of games used in rehabilitation programs should be constantly improved. Program updates with the ability to get new games would contribute to maintaining clinical progress. 

## 5. Conclusions

Fully immersive virtual reality therapy with head-mounted displays (HMD) appears to be beneficial for patients with upper limb complications after a stroke. Despite the small amount of research, the therapy seems to be promising, affecting the regeneration of the upper limb. In order to clearly prove the positive effects of VR immersion therapy, a larger number of studies should be performed with a larger research sample and a longer study time. 

## Figures and Tables

**Table 1:** Brief description of the studies included in the review.

Study, Year, References	Type of Study	Duration of the Study	Results	Conclusions	Other
Ögün et al., 2019 [44].	Randomized, controlled, double-blinded study	6 weeks	The results of the paired-samples *t*-test showed that FMUE, ARAT, FIM and PASS scores increased significantly compared to baseline values in the VR group (*p* < 0.001). FMUE (*p* < 0.001), ARAT (*p* < 0.001), and FIM (*p* = 0.002) scores increased significantly in the control group. Differences in PASS-BADL (*p* = 0.509) and PASS-IADL (*p* = 0.542) scores were not significant. Comparing the differences between posttest and pretest values of all outcome measures (FMUE, FIM, ARAT, PASS-BADL, and PASS-IADL) of patients in both groups, there was a significant difference in favor of the VR group (*p* < 0.001).	Immersive VR rehabilitation proved effective in improving upper limb function and self-care skills but did not improve functional independence.	Number of patients in the control group: 32 Number of patients in the study group: 33 Study discontinued: 10 patients in the research group and 9 patients in the control group.
Weber et al., 2019 [45].	Pilot study	4 weeks	Fugl-Meyer motor scores showed a slight improvement for the upper limb from 21.7 (SD = 8.68) to 22.8 (SD = 9.19), which did not reach statistical significance (*p* = 0.084). A t-test for paired samples to compare ARAT test results observed no significant difference from baseline (M = 9.1, SD=8.05) to posttest (M = 9.8, SD = 9.08); *p* = 0.33).	Chronic stroke patients well tolerated four weeks of immersive virtual reality mirror therapy.	One patient withdrew from the study. The main objective of the study was the feasibility of immersive virtual reality mirror therapy for post-stroke upper limb paresis.
Lee et al., 2020 [46].	Feasibility study	4 weeks	Of the nine patients who completed the training, five showed improvement both in ARAT and BBT. ARAT (pre-training 22.3 ± 20.1, post-training 31.1 ± 19.6; *p* = 0.028), BBT (pre-training 11.2 ± 16.3, post-training 19.6 ± 29.3; *p* =.012), and MBI (pre-training 90.4 ± 8.5, post-training 93.0 ± 5.0; *p* = 0.042) significantly improved after the training.	Immersive VR therapy may positively improve upper limb function and activities of daily living in post-stroke patients without serious adverse effects.	The aim of the study was to investigate not only the efficacy of the therapy, but also the usefulness and feasibility of a rehabilitation program. Three patients discontinued the study. The authors also examined usability using a questionnaire with a 7-point Likert scale
Huang et al., 2020 [47].	Single-blind clinical trial, pretest–posttest control group design trial	8 weeks	Both groups were significantly differences in FMA (Conventional group, *p* = 0.021; Immersive virtual reality group, *p* = 0.014)	Immersive virtual reality gaming device contributes to improving upper limb function.	
Mekbib et al., 2020 [48].	Controlled clinical trial	2 weeks	After the intervention, an improvement in FM-UE score was observed for 2 weeks only (*p* < 0.042; Cohen’s effective size d = 0.7, moderate effect).	Unilateral and bilateral limb mirroring exercises in an immersive virtual environment can lead to improved motor function.	The study included 12 patients in the study group from 15 in the control group. In the study, the authors also examined cortical reorganization using magnetic resonance imaging (MRI). They proved that exercise can improve cortical reorganization.
Mekbib et al., 2021 [49].	Clinical, randomized with a single-parallel blind study.	2 weeks	The research group significantly improved the results of FM-EU (*p* = 0.0001) and BI (*p* = 0.003) from the beginning to the end of the intervention. The control group also improved significantly in BI (*p* = 0.011) from baseline to end of intervention, but not in FM-EU results (*p* = 0.072). There was a significant difference between the two groups in terms of FM-EU (*p* = 0.007, d. Cohen = 0.7) after the intervention, indicating that the research group improved the FM-EU values moderately better than the control group. In terms of BI results, no significant difference was observed between the groups (*p* = 0. 193).	After rehabilitation with immersion therapy, motor impairments and functional independence of the patients were reduced.	A total of 28 patients met the inclusion criteria. The control group consisted of 15 patients. Two patients from the research group and three patients from the control group withdrew during the study. The authors also studied the stimulation and activation of mirror neurons using MRI, based on unilateral and bilateral upper limb exercises in an immersive environment.
Hsu et al., 2022 [50].	Single-blinded, randomized, controlled trial.	12 weeks	Statistically significant group interaction effects were observed for the wrist subscale in FM-UE (GEE, *p* = 0.012) and BBT (GEE, *p* = 0.044). GEE—generalized estimating equations method with unstructured variance and covariance matrix.	Mirror immersive VR therapy has the potential to restore upper limb motor function in post-stroke patients.	Two patients discontinued the study. The study lasted 9 weeks, but patients were evaluated after 12 weeks.

**Table 2:** Detailed description of the studies included in the review.

Study, Year, References	Type of Stroke	Number of Patients Tested	Research Instruments: Scales, Tests	Research Group vs. Control Group
Ögün et al., 2019 [44].	Ischemic stroke	65	Action Research Arm Test (ARAT), Functional Independence Measure (FIM), Fugl-Meyer Upper Extremity Scale (FMUE), Performance Assessment of Self-Care Skills (PASS).	Rehabilitation consisted of 18 therapy sessions three days a week for six weeks. Research group—60 min immersion rehabilitation program of the upper extremity VR. Control group—45 min of conventional therapy, 15 min of sham VR program.
Weber et al., 2019 [45].	Ischemic and hemorrhagic stroke	11	Fugl-Meyer Upper Extremity, Action Research Arm Test.	Twelve program sessions of 30 min each. The study did not have a control group. All patients performed the same exercises
Lee et al., 2020 [46].	Ischemic and hemorrhagic stroke	12	Action Research Arm Test, Box and Block Test (BBT), Modified Barthel Index (MBI), Self-reported questionnaire.	Participants performed a total of 10 sessions two to three times a week, 30 min per session. The study did not have a control group. All patients performed the same exercises.
Huang et al., 2020 [47].	Not specified	18	Fugl-Meyer Assessment (FMA), Box and Block Test, Functional Independence Measure.	Research group: over the course of 8 weeks, patients had 20 sessions to complete. There were 3 training sessions of 30 min each in 1 week, 60 min conventional training—a total of 30 h. Control Group: Conventional training of the upper extremities for 90 min—20 sessions—a total of 30 h.
Mekbib et al., 2020 [48].	Ischemic and hemorrhagic stroke	12	Fugl-Meyer Upper Extremity (FM-UE).	Research group: training 8 h of VR and 8 h of conventional therapy over 2 weeks (1 h of VR and 1 h of conventional therapy per day for 4 days per week)
Mekbib et al., 2021 [49].	Ischemic and hemorrhagic stroke	43	Fugl-Meyer Upper Extremity, Barthel Index (BI).	Research group: 1 h VR and 1 h occupational therapy (OT) daily, 4 days a week for 2 weeks. Control group: occupational therapy 2 h per day, 4 days per week for 2 weeks.
Hsu et al., 2022 [50].	Ischemic and hemorrhagic stroke	54	Fugl-Meyer Upper Extremities, Box and Block Test, Semmes–Weinstein monofilament, Motor activity log, Modified Ashworth scale (MAS)	Each patient performed 20 min of exercise, then depending on the group: COT (conventional, occupational therapy), MT (mirror therapy), or VR-MT (immersive VR-based mirror therapy), they performed 30 min of their program twice a week for 9 weeks.

## Data Availability

Not applicable.
